# Effectiveness of surgery and hyperbaric oxygen for antiresorptive agent-related osteonecrosis of the jaw: A subgroup analysis by disease stage

**DOI:** 10.1371/journal.pone.0244859

**Published:** 2021-01-04

**Authors:** Takuma Watanabe, Keita Asai, Shizuko Fukuhara, Ryuji Uozumi, Kazuhisa Bessho

**Affiliations:** 1 Department of Oral and Maxillofacial Surgery, Graduate School of Medicine, Kyoto University, Kyoto, Japan; 2 Department of Biomedical Statistics and Bioinformatics, Graduate School of Medicine, Kyoto University, Kyoto, Japan; Thamar University, Faculty of Dentistry, YEMEN

## Abstract

Antiresorptive agent-related osteonecrosis of the jaw (ARONJ) is an adverse event induced by antiresorptive agents (ARAs). The purpose of this study was to evaluate variables, mainly surgery and hyperbaric oxygen (HBO) therapy, associated with treatment outcomes in patients with a diagnosis of ARONJ at a single center. We enrolled consecutive patients who presented to our hospital for the management of stage 2 or 3 ARONJ between January 2003 and December 2019. The relationship between potentially predictive factors and outcome variables was examined using statistical analyses, along with a subgroup analysis based on disease stage. Of 252 patients included in this study, 206 had stage 2 ARONJ and 46 had stage 3 ARONJ. There were 119 patients with osteoporosis and 133 with malignant disease. In total, 139 patients were healed, and the healing rate of patients with stage 3 ARONJ was lower than that of patients with stage 2 ARONJ. With regard to the combination of surgery and HBO therapy, most patients underwent HBO before and after surgery. In the univariable analysis, surgery showed a therapeutic effect in both stage 2 and 3 ARONJ, whereas HBO showed a therapeutic effect in stage 2 ARONJ. In the multivariable analysis for stage 2 ARONJ, extensive surgery showed a stronger association with healing than conservative surgery, whereas ≥46 sessions of HBO therapy was less associated with healing than was non-HBO therapy. Our findings suggest that extensive surgery is highly effective against ARONJ regardless of disease stage if there is a sequestrum separation and systemic tolerance, whereas HBO therapy before and after surgical approach can be effective. Further studies are needed to identify treatment strategies for patients with treatment-refractory ARONJ who may be forced to undergo long-term HBO therapy with the expectation of sequestrum separation.

## Introduction

Antiresorptive agent-related osteonecrosis of the jaw (ARONJ) is an adverse event that is induced by antiresorptive agents (ARAs), such as bisphosphonate (BP) and denosumab (Dmab), as well as angiogenesis inhibitors. Antiresorptive agent-related osteonecrosis of the jaw can significantly impair a patient’s quality of life. The first cases of BP-related osteonecrosis of the jaw (BRONJ) were described by Marx in 2003 [1]. Later in 2011, cases of Dmab-related osteonecrosis of the jaw (DRONJ) were reported during randomized clinical trials [2]. Although clinical and basic research regarding ARONJ have been performed to clarify its pathophysiological mechanisms, the reasons why this condition occurs in patients taking ARAs remain unknown [3].

The best treatment practices for the management of patients with ARONJ are also controversial since the pathophysiological mechanisms are unclear. Management of ARONJ includes a wide variety of strategies. Nonsurgical treatment includes antimicrobial mouth rinses, systemic antibiotics, hyperbaric oxygen (HBO) therapy, pentoxifylline, and teriparatide. Surgical treatment includes curettage, sequestrectomy, debridement, saucerization, and surgical resection [4]. Recently, several clinical studies have reported that a surgical approach is more successful than a nonsurgical approach for the treatment of ARONJ [4–9].

Hyperbaric oxygen therapy has been investigated as a potentially useful adjunct therapy (with a surgical approach and systemic antibiotic therapy) for pain relief and early healing, particularly in more severe cases [10, 11]. Nevertheless, the effectiveness of HBO therapy in patients with ARONJ has not been evaluated in clinical studies with large sample sizes. Therefore, the purpose of this study was to evaluate the clinical outcomes for variables, mainly surgery and HBO therapy, in patients with ARONJ. We planned to examine patient records from 2003 to 2019 and perform a subgroup analysis by disease stage to assess differences between stage 2 and 3 ARONJ.

## Materials and methods

### Patients

We designed and implemented a single-center cohort study. The study population included all consecutive patients who had presented to the Department of Oral and Maxillofacial Surgery at Kyoto University Hospital for management of ARONJ, between January 2003 and December 2019. Diagnosis of ARONJ was based on the criteria in the Position Paper [12, 13]. Clinical staging of ARONJ was based on the same position paper [12, 13]. We included patients with stage 2 or 3 ARONJ at the initial visit and who had been followed up for a minimum of three months. Patients with insufficient data on study variables in their medical records were excluded.

### Variables

Potentially predictive variables from the patients’ medical records were examined. These were categorized as 1) demographic, 2) clinical, 3) treatment-related, and 4) history of ARA use. Demographic variables included sex and age at the time of ARONJ diagnosis. Clinical variables included anatomic location of the exposed bone (maxilla, mandible, or both), initiating event (tooth extraction, periodontitis, ill-fitting prosthesis, mucosal ulceration, peri-implantitis, or spontaneous), disease stage (2 or 3), and comorbidities (hypertension, diabetes mellitus, conditions requiring steroid therapy, or cancer requiring chemotherapy). Treatment-related variables included surgery and HBO performed based on the condition of ARONJ. Surgery was divided into conservative (removal of only necrotic bone) and extensive (removal of necrotic and surrounding bone until bleeding is observed in almost all areas, marginal mandibulectomy, or segmental mandibulectomy) surgery. We performed surgery only in cases where it was not contraindicated by the patient’s general health status. Extensive surgery was only performed if clinical and imaging findings indicated a sequestrum separation; else, conservative surgery was performed. Primary wound closure was performed in almost all patients who underwent extensive surgery. Hyperbaric oxygen therapy was administered using a Kawasaki KHO-302A multi-place chamber (Kawasaki Engineering Corp., Kobe, Japan), with 100% oxygen at 2.0 atmospheres absolute. It was set up as one cycle of 15 sessions (80 minutes per session) and used for several cycles depending on the disease condition, unless there were contraindications, such as obstructive pulmonary disease or middle ear problems. Patients receiving HBO were typically required to stay in the hospital as part of our protocol. Variables related to the history of ARA use included the following: indication for ARA (osteoporosis on low-dose therapy or malignant disease on high-dose therapy), type and duration of therapy (duration of BP for BRONJ or duration of Dmab for DRONJ), and ARA discontinuation. Classification of low-dose and high-dose therapy was based on a previous review [14]. The distinction between BRONJ and DRONJ was based on the type of ARA used at the time of the initial visit. Other treatment modalities, including oral care, antimicrobial mouthwash, systemic antibiotic therapy, and local irrigation, were used in almost all patients and were not included as study variables. With regard to antibiotics, the regimen was adjusted in almost all patients according to the results of the microbial culture. Ampicillin/sulbactam, amoxicillin/clavulanate, and metronidazole were mainly used.

Outcomes were divided into four categories: healed, improved, stable, or worse. These categories were subsequently dichotomized as healed or improved/stable/worse. Patients were considered healed if there was complete mucosalization over previously exposed bone. Patients were considered improved if there was downstaging, stable if there was no change in stage, and worse if there was upstaging.

### Statistical analysis

We performed a subgroup analysis on the basis of disease stage. All statistical analyses were performed using JMP^®^ 14 (SAS Institute, Cary, NC, USA). First, the univariable analysis between each variable (including surgery and HBO therapy) and outcome variable was performed. The chi-square test was used to compare categorical variables and the Student’s *t*-test to compare continuous variables. Second, multivariable logistic regression for selected variables (with reference to healed status) was performed to calculate the odds ratio (OR) and 95% confidence interval (CI). The variables included in the multivariable logistic model were selected on the basis of the expert knowledge of oral and maxillofacial surgeons and the results of the univariable analysis.

### Ethical approval

Informed consent was obtained in the form of opt-out on the homepage of the hospital website. This study was approved by the institutional review board of Kyoto University Hospital (R0213-4).

## Results

A summary of patient characteristics is presented in Table 1. A total of 252 patients with ARONJ were included in this study. There were 206 patients with stage 2 ARONJ and 46 with stage 3 ARONJ. Most patients were female (n = 196, 77.8%), and the overall mean age was 71.2 ± 9.9 years. Antiresorptive agent-related osteonecrosis of the jaw was most frequently located in the mandible (n = 137, 54.4%), and the most frequent initiating event was tooth extraction (n = 114, 45.2%). Of the 252 patients, 138 (54.8%) underwent surgery and 143 (56.7%) received HBO therapy. There were 119 (47.2%) patients with osteoporosis and 133 (52.8%) with malignant disease. The most common type of ARONJ in this study was BRONJ (n = 206, 81.7%). Most patients continued ARA therapy (n = 213, 84.5%). The most frequent comorbidity was hypertension (n = 109, 43.3%). The percentage of patients with stage 3 ARONJ undergoing ≥46 sessions of HBO therapy (n = 13, 28.3%) was higher than that of patients with stage 2 ARONJ (n = 18, 8.7%).

**Table 1:** Characteristics of the study patients.

Variable			Total cases (n = 252)		Stage 2 (n = 206)		Stage 3 (n = 46)		*p* value
Sex									0.383^a^
	Male		56	(22.2)	48	(23.3)	8	(17.4)	
	Female		196	(77.8)	158	(76.7)	38	(82.6)	
Age (years)									0.218^b^
	Range		38–95		38–95		52–91		
	Mean±standard deviation		71.2±9.9		70.8±9.8		72.8±10.2		
Anatomic location									0.252^a^
	Maxilla		75	(29.7)	64	(31.1)	11	(23.9)	
	Mandible		137	(54.4)	107	(51.9)	30	(65.2)	
	Maxilla and mandible		40	(15.9)	35	(17.0)	5	(10.9)	
Initiating event									0.357^a^
	Tooth extraction		114	(45.2)	96	(46.6)	18	(39.1)	
	Periodontitis		86	(34.1)	69	(33.5)	17	(37.0)	
	Ill-fitting prosthesis (denture trauma)		15	(6.0)	14	(6.8)	1	(2.2)	
	Mucosal ulceration		10	(4.0)	9	(4.4)	1	(2.2)	
	Peri-implantitis		3	(1.2)	3	(1.4)	0	(0.0)	
	Spontaneous (other)		24	(9.5)	15	(7.3)	9	(19.5)	
Surgery									0.542^a^
	(-)		114	(45.2)	95	(46.1)	19	(41.3)	
	Conservative		46	(18.3)	39	(18.9)	7	(15.2)	
	Extensive		92	(36.5)	72	(35.0)	20	(43.5)	
HBO									0.004^a^
	(-)		109	(43.3)	95	(46.1)	14	(30.4)	
	1–15 sessions		22	(8.7)	20	(9.7)	2	(4.3)	
	16–30 sessions		50	(19.8)	42	(20.4)	8	(17.4)	
	31–45 sessions		40	(15.9)	31	(15.1)	9	(19.6)	
	≥46 sessions		31	(12.3)	18	(8.7)	13	(28.3)	
Indication for ARA									0.676^a^
	Osteoporosis		119	(47.2)	96	(46.6)	23	(50.0)	
	Malignant disease		133	(52.8)	110	(53.4)	23	(50.0)	
Type and duration of ARA therapy									0.272^a^
	BRONJ		206	(81.7)	171	(83.0)	35	(76.1)	
		Bisphosphonate							
		Median(interquartile Range)	36.0(20.0–62.5)		36.0(20.0–58.0)		39.0(22.0–112.0)		
	DRONJ		46	(18.3)	35	(17.0)	11	(23.9)	
		Denosumab							
		Median(interquartile Range)	14.0(7.0–30.3)		12.0(6.0–22.0)		28.0(14.0–46.0)		
ARA discontinuation									0.957^a^
	No		213	(84.5)	174	(84.5)	39	(84.8)	
	Yes		39	(15.5)	32	(15.5)	7	(15.2)	
Comorbidities^c^									
	Hypertension								0.489^a^
		(-)	143	(56.7)	119	(57.8)	24	(52.2)	
		(+)	109	(43.3)	87	(42.2)	22	(47.8)	
	Diabetes mellitus								0.011^a^
		(-)	212	(84.1)	179	(86.9)	33	(71.6)	
		(+)	40	(15.9)	27	(13.1)	13	(28.4)	
	Conditions requiring steroid therapy								0.715^a^
		(-)	192	(76.2)	156	(75.7)	36	(78.3)	
		(+)	60	(23.8)	50	(24.3)	10	(21.7)	
	Cancer requiring chemotherapy								0.118^a^
		(-)	185	(73.4)	147	(71.4)	38	(82.6)	
		(+)	67	(26.6)	59	(28.6)	8	(17.4)	

Data are presented as n (%) unless otherwise indicated.

HBO = hyperbaric oxygen; ARA = antiresorptive agent; BRONJ = bisphosphonate-related osteonecrosis of the jaw; DRONJ = denosumab-related osteonecrosis of the jaw.

^a^ Chi-square test.

^b^ Student’s t-test.

^c^ Patients may have more than one comorbidity.

The treatment outcome of patients with ARONJ is presented in Table 2. A total of 139 patients (55.2%) were healed. Of the 138 patients who underwent surgery, and the 143 patients who received HBO therapy, 116 (84.1%) and 97 (67.8%) patients were healed, respectively. The healing rate of patients with stage 3 ARONJ (n = 23, 50.0%) was lower than that of patients with stage 2 ARONJ (n = 116, 56.3%). The treatment outcome of patients who underwent a combination of surgery and HBO therapy is presented in Table 3. Of the 109 patients, 105 underwent HBO therapy before and after surgery, of which 88 (83.8%) were healed. In patients undergoing both surgery and HBO therapy, the healing rate for stage 3 ARONJ (n = 20, 87.0%) was higher than that for stage 2 ARONJ (n = 70, 81.4%).

**Table 2:** Treatment outcome of patients with ARONJ.

			Healed		Improved		Stable		Worse	
Total cases (n = 252)			139	(55.2)	61	(24.2)	50	(19.8)	2	(0.8)
	Surgery									
		(-) (n = 114)	23	(20.2)	44	(38.6)	45	(39.5)	2	(1.7)
		Conservative (n = 46)	39	(84.8)	6	(13.0)	1	(2.2)	0	(0.0)
		Extensive (n = 92)	77	(83.7)	11	(12.0)	4	(4.3)	0	(0.0)
	HBO									
		(-) (n = 109)	42	(38.5)	34	(31.2)	32	(29.4)	1	(0.9)
		1–15 sessions (n = 22)	14	(63.6)	4	(18.2)	3	(13.6)	1	(4.6)
		16–30 sessions (n = 50)	36	(72.0)	9	(18.0)	5	(10.0)	0	(0.0)
		31–45 sessions (n = 40)	31	(77.5)	6	(15.0)	3	(7.5)	0	(0.0)
		≥46 sessions (n = 31)	16	(51.6)	8	(25.8)	7	(22.6)	0	(0.0)
Stage 2 (n = 206)			116	(56.3)	56	(27.2)	32	(15.5)	2	(1.0)
	Surgery									
		(-) (n = 95)	23	(24.2)	43	(45.3)	27	(28.4)	2	(2.1)
		Conservative (n = 39)	33	(84.6)	5	(12.8)	1	(2.6)	0	(0.0)
		Extensive (n = 72)	60	(83.3)	8	(11.1)	4	(5.6)	0	(0.0)
	HBO									
		(-) (n = 95)	39	(41.1)	32	(33.7)	23	(24.2)	1	(1.0)
		1–15 sessions (n = 20)	13	(65.0)	4	(20.0)	2	(10.0)	1	(5.0)
		16–30 sessions (n = 42)	31	(73.8)	8	(19.1)	3	(7.1)	0	(0.0)
		31–45 sessions (n = 31)	25	(80.6)	6	(19.4)	0	(0.0)	0	(0.0)
		≥46 sessions (n = 18)	8	(44.5)	6	(33.3)	4	(22.2)	0	(0.0)
Stage 3 (n = 46)			23	(50.0)	5	(10.9)	18	(39.1)	0	(0.0)
	Surgery									
		(-) (n = 19)	0	(0.0)	1	(5.3)	18	(94.7)	0	(0.0)
		Conservative (n = 7)	6	(85.7)	1	(14.3)	0	(0.0)	0	(0.0)
		Extensive (n = 20)	17	(85.0)	3	(15.0)	0	(0.0)	0	(0.0)
	HBO									
		(-) (n = 14)	3	(21.4)	2	(14.3)	9	(64.3)	0	(0.0)
		1–15 sessions (n = 2)	1	(50.0)	0	(0.0)	1	(50.0)	0	(0.0)
		16–30 sessions (n = 8)	5	(62.5)	1	(12.5)	2	(25.0)	0	(0.0)
		31–45 sessions (n = 9)	6	(66.7)	0	(0.0)	3	(33.3)	0	(0.0)
		≥46 sessions (n = 13)	8	(61.5)	2	(15.4)	3	(23.1)	0	(0.0)

Data are presented as n (%) unless otherwise indicated.

ARONJ = Antiresorptive agent-related osteonecrosis of the jaw; HBO = hyperbaric oxygen.

**Table 3:** Treatment outcome of patients with a combination of surgery and HBO.

		Healed		Improved		Stable		Worse	
Total cases (n = 109)		90	(82.6)	15	(13.8)	4	(3.6)	0	(0.0)
	HBO only before Surgery (n = 2)	1	(50.0)	1	(50.0)	0	(0.0)	0	(0.0)
	HBO only after Surgery (n = 2)	1	(50.0)	0	(0.0)	1	(50.0)	0	(0.0)
	HBO before and after Surgery (n = 105)	88	(83.8)	14	(13.3)	3	(2.9)	0	(0.0)
Stage 2 (n = 86)		70	(81.4)	12	(14.0)	4	(4.6)	0	(0.0)
	HBO only before Surgery (n = 1)	1	(100.0)	0	(0.0)	0	(0.0)	0	(0.0)
	HBO only after Surgery (n = 2)	1	(50.0)	0	(0.0)	1	(50.0)	0	(0.0)
	HBO before and after Surgery (n = 83)	68	(81.9)	12	(14.5)	3	(3.6)	0	(0.0)
Stage 3 (n = 23)		20	(87.0)	3	(13.0)	0	(0.0)	0	(0.0)
	HBO only before Surgery (n = 1)	0	(0.0)	1	(100.0)	0	(0.0)	0	(0.0)
	HBO only after Surgery (n = 0)	0	(0.0)	0	(0.0)	0	(0.0)	0	(0.0)
	HBO before and after Surgery (n = 22)	20	(90.9)	2	(9.1)	0	(0.0)	0	(0.0)

Data are presented as n (%) unless otherwise indicated.

HBO = hyperbaric oxygen.

Univariable analysis showed strong associations between treatment outcomes and sex (*p* = 0.045), anatomic location (*p* = 0.023), surgery (*p*<0.001), HBO therapy (*p*<0.001), indication for ARA (*p*<0.001), ARA type (*p* = 0.001), hypertension (*p* = 0.002), and cancer requiring chemotherapy (*p*<0.001) in stage 2 ARONJ. In stage 3 ARONJ, strong associations between outcomes and surgery (*p*<0.001), indication for ARA (*p* = 0.001), and ARA type (*p* = 0.016) were observed. Healing was not observed in patients who did not undergo surgery (Table 4). Multivariable logistic regression showed that healed status was associated with conservative surgery (OR, 14.046; 95% CI, 4.035–48.897; *p*<0.001), extensive surgery (OR, 51.830; 95% CI, 11.481–233.983; *p*<0.001), ≥46 sessions of HBO therapy (OR, 0.121; 95% CI, 0.019–0.782; *p* = 0.027), and indication for ARA (OR, 0.229; 95% CI, 0.075–0.695; *p* = 0.009) in stage 2 ARONJ. In stage 3 ARONJ, variables of surgery could not be selected as covariates because of the number of events, whereas healed status was associated with indication for ARA (OR, 0.145; 95% CI, 0.034–0.544; *p* = 0.004) (Table 5).

**Table 4:** Univariable analysis of study variables by treatment outcome.

Characteristic			Total cases (n = 252)			Stage 2 (n = 206)			Stage 3 (n = 46)		
			Healed	Others	*p* value	Healed	Others	*p* value	Healed	Others	*p* value
			(n = 139)	(n = 113)		(n = 116)	(n = 90)		(n = 23)	(n = 23)	
Sex					0.016^a^			0.045^a^			0.120^a^
	Male		23	33		21	27		2	6	
	Female		116	80		95	63		21	17	
Age(years)					0.525^b^			0.278^b^			0.539^b^
	Mean±Standard Deviation		71.5±9.8	70.7±9.9		71.5±10.0	70.0±9.5		71.9±9.3	73.7±11.1	
Anatomic location					0.035^a^			0.023^a^			0.865^a^
	Maxilla		41	34		35	29		6	5	
	Mandible		83	54		68	39		15	15	
	Maxilla and mandible		15	25		13	22		2	3	
Initiating event					0.070^a^			0.094^a^			0.546^a^
	Tooth extraction		70	44		60	36		10	8	
	Other		69	69		56	54		13	15	
Surgery					<0.001^a^			<0.001^a^			<0.001^a^
	(-)		23	91		23	72		0	19	
	Conservative		39	7		33	6		6	1	
	Extensive		77	15		60	12		17	3	
HBO					<0.001^a^			<0.001^a^			0.149^a^
	(-)		42	67		39	56		3	11	
	1–15 sessions		14	8		13	7		1	1	
	16–30 sessions		36	14		31	11		5	3	
	31–45 sessions		31	9		25	6		6	3	
	≥46 sessions		16	15		8	10		8	5	
Indication for ARA					<0.001^a^			<0.001^a^			0.001^a^
	Osteoporosis		93	26		76	20		17	6	
	Malignant disease		46	87		40	70		6	17	
ARA type					<0.001^a^			0.001^a^			0.016^a^
	BRONJ		126	80		105	66		21	14	
	DRONJ		13	33		11	24		2	9	
ARA discontinuation					0.384^a^			0.248^a^			0.681^a^
	No		115	98		95	79		20	19	
	Yes		24	15		21	11		3	4	
Comorbidities											
	Hypertension				0.002^a^			0.002^a^			0.555^a^
		(-)	67	76		56	63		11	13	
		(+)	72	37		60	27		12	10	
	Diabetes mellitus				0.746^a^			0.455^a^			0.743^a^
		(-)	116	96		99	80		17	16	
		(+)	23	17		17	10		6	7	
	Conditions requiring steroid therapy				0.223^a^			0.173^a^			1.000^a^
		(-)	110	82		92	64		18	18	
		(+)	29	31		24	26		5	5	
	Cancer requiring chemotherapy				<0.001^a^			<0.001^a^			0.120^a^
		(-)	119	66		98	49		21	17	
		(+)	20	47		18	41		2	6	

Data are presented as n.

HBO = hyperbaric oxygen; ARA = antiresorptive agent; BRONJ = bisphosphonate-related osteonecrosis of the jaw; DRONJ = denosumab-related osteonecrosis of the jaw.

^a^ Chi-square test.

^b^ Student's t-test.

**Table 5:** Multivariable logistic regression analysis of variables relating to healed status.

Characteristic		Total cases			Stage 2			Stage 3		
		OR	95% CI	*p* value	OR	95% CI	*p* value	OR	95% CI	*p* value
Sex										
	Male	Reference	-	-	Reference	-	-			
	Female	0.703	0.277–1.789	0.460	0.450	0.257–1.828	0.460			
Anatomic location										
	Maxilla	Reference	-	-	Reference	-	-			
	Mandible	2.046	0.827–5.063	0.121	2.138	0.835–5.473	0.113			
	Maxilla and mandible	0.514	0.154–1.721	0.281	0.472	0.131–1.701	0.251			
Surgery										
	(-)	Reference	-	-	Reference	-	-			
	Conservative	18.071	5.723–57.064	<0.001	14.046	4.035–48.897	<0.001			
	Extensive	64.584	17.057–244.546	<0.001	51.830	11.481–233.983	<0.001			
HBO										
	(-)	Reference	-	-	Reference	-	-			
	1–15 sessions	2.584	0.561–11.901	0.223	2.086	0.450–9.674	0.348			
	16–30 sessions	0.826	0.241–2.827	0.761	0.915	0.248–3.370	0.761			
	31–45 sessions	0.957	0.257–3.558	0.948	0.788	0.178–3.481	0.753			
	≥46 sessions	0.170	0.035–0.819	0.027	0.121	0.019–0.782	0.027			
Indication for ARA										
	Osteoporosis	Reference	-	-	Reference	-	-	Reference	-	-
	Malignant disease	0.200	0.072–0.561	0.002	0.229	0.075–0.695	0.009	0.145	0.034–0.544	0.004
ARA type										
	BRONJ	Reference	-	-	Reference	-	-	Reference	-	-
	DRONJ	0.376	0.128–1.103	0.075	0.494	0.151–1.615	0.243	0.192	0.024–1.044	0.057
Hypertension										
	(-)	Reference	-	-	Reference	-	-			
	(+)	1.140	0.509–2.553	0.749	1.229	0.518–2.918	0.640			
Cancer requiring chemotherapy										
	(-)	Reference	-	-	Reference	-	-			
	(+)	0.522	0.187–1.454	0.214	0.539	0.183–1.587	0.262			

HBO = hyperbaric oxygen; ARA = antiresorptive agent; BRONJ = bisphosphonate-related osteonecrosis of the jaw; DRONJ = denosumab-related osteonecrosis of the jaw; OR = odds ratio; CI = confidence interval.

## Discussion

In this single-center cohort study, we evaluated the association of surgical and HBO treatments with clinical outcomes in patients with ARONJ, along with a subgroup analysis based on the disease stage; this included the type of surgery and the timing and number of HBO therapy sessions, which are important clinical considerations. In the univariable analysis, surgery showed a therapeutic effect in both stage 2 and 3 ARONJ, while HBO only showed a therapeutic effect in stage 2 ARONJ. In the multivariable analysis for stage 2 ARONJ, extensive surgery displayed a stronger association with healing than conservative surgery, while ≥46 sessions of HBO therapy was less associated with healing than non-HBO therapy.

Although several previous studies on surgical treatment included patients with stage 1 ARONJ [4, 6, 7, 9], the American Association of Oral and Maxillofacial Surgeons (AAOMS) reports that patients with stage 1 ARONJ benefit from medical management, including the use of oral antimicrobial rinses, and no immediate operative treatment is required [12]. The AAOMS has also suggested a stage-specific approach for treatment, in which medical management is indicated for the earlier stages (stage 0 and 1), and surgical interventions are preferred for the later stages (stage 2 and 3) [12, 15]. To accurately investigate the effectiveness of surgery according to the stage-specific approach, we limited inclusion to patients with stage 2 or 3 ARONJ in the present study.

Diseases that affect bone, such as osteoporosis or bone malignancies, can have debilitating effects on patients’ lives by causing fractures and other complications [14]. Low-dose BP or Dmab are used for the prevention and treatment of osteoporosis, which occurs in postmenopausal women and men and subsequent to glucocorticoid use [14, 16]. In contrast, high-dose BP or Dmab is used to maintain health-related quality of life in patients with bone metastasis and to reduce the risk of skeletal complications caused by multiple myeloma [14, 17]. In accordance with the findings of previous studies, in this study, the number of patients with malignant disease on high-dose ARA therapy was greater than those with osteoporosis on low-dose ARA therapy [4, 6, 7, 9]. Multivariable analysis also showed that osteoporosis and malignant disease can have an impact on the outcome of ARONJ.

Although there are no universally accepted treatment protocols for ARONJ, palliation of symptoms and control of associated infection are generally accepted approaches [18]. The AAOMS suggests that treatment objectives for patients with ARONJ are to 1) eliminate pain; 2) control infection of the soft and hard tissues; and 3) minimize the progression or occurrence of bone necrosis [12]. The majority of patients with ARONJ are managed conservatively according to the previous recommendations of the Canadian Association of Oral and Maxillofacial Surgeons, AAOMS, and American Dental Association, and this approach is supported by many practitioners [12, 19, 20]. One task force recommended conservative therapy, as long as there is no 1) obvious progression of disease; 2) pain that is not controlled with conservative means; or 3) discontinuation of antiresorptive therapy on the advice of the oncologist [16]. For the conservative approach, there is a consensus that antibiotic treatment is needed in all patients with ARONJ stage 2 or 3 [12, 21–23]. A previous literature review reported that the most common perioperative antibiotic regimen is penicillin-based antibiotics plus ꞵ-lactamase inhibitor or metronidazole, with variation in the duration of administration [24]. In our study, these antibiotics were mainly used according to the results of microbial culture, in which infected bone or infectious granulation tissue samples were collected. The duration of administration varied from patient to patient depending on the extent of infection.

A previous systematic review reported that outcomes for every ARONJ stage were relatively poor when patients were treated with nonsurgical therapies [25]. The authors also concluded that a surgical approach to ARONJ lesions seemed to be generally more effective for every disease stage [25]. Furthermore, a recent multi-center retrospective study indicated that extensive surgery was superior to both conservative surgery and nonsurgical therapy in the treatment of patients with ARONJ [9]. Consistent with previous studies [4–9], patients in the present study who underwent surgical treatment, particularly extensive surgery, had a favorable outcome.

For stage-specific treatment, the AAOMS suggests that conservative surgery directed at reducing the volume of colonized necrotic bone may be beneficial in patients with stage 2 ARONJ, and that patients with stage 3 ARONJ benefit from conservative or extensive surgery [12]. We generally performed extensive surgery whenever possible regardless of disease stage if clinical and imaging findings indicated a sequestrum separation. That is, the decision criteria for extensive surgery included not only disease stage but also sequestrum separation. In fact, there was no major difference in the percentage of the type of surgery performed for stage 2 and 3 ARONJ. Although the healing rate was similar for both conservative surgery and extensive surgery, extensive surgery was more associated with healing than conservative surgery after adjusting for prognostic factors. Systemic background, such as malignant disease condition or life prognosis, can affect the application of non-surgery, conservative surgery, or extensive surgery. We believe that extensive surgery is generally effective.

Hyperbaric oxygen therapy produces reactive nitrogen species and reactive oxygen species, which influence osteoclast differentiation and activity; therefore, it participates in the regulation of various aspects of bone metabolism [26–28]. Although treatment with HBO has long been associated solely with the treatment of osteoradionecrosis [29, 30], the potential effects of HBO on ARONJ have not been fully investigated. Freiberger et al. hypothesized in their randomized controlled trial that signaling for bone turnover might be augmented by production of reactive nitrogen species and reactive oxygen species and concluded that HBO appeared to be a useful adjunct to ARONJ treatment, particularly for more severe cases [11]. Furthermore, the first systematic review of oxygen therapy for the management of ARONJ reported that it was difficult to determine whether HBO therapy is superior to the placebo due to the absence of high-level evidence in the literature; the authors reported only two case reports [31, 32], three case series [10, 33, 34], and a single randomized controlled trial [11].

The present study is unique in that it included 143 patients who underwent HBO therapy; till date, this is the largest sample size among clinical studies, which have evaluated HBO treatment for ARONJ. Furthermore, to our best knowledge, this seems to be the first study to address the timing or number of HBO therapy sessions. The large sample size may be attributed to the fact that many patients with ARONJ are referred to our institution, which has large hyperbaric chambers for therapeutic purposes. Indeed, no other nearby facilities currently offer HBO therapy. Since HBO therapy is not an invasive procedure and has a relatively wide range of applications, it can be considered an effective adjunctive treatment for ARONJ to achieve anti-inflammation and sequestrum separation.

We acknowledge that there were several limitations to this study. First, its retrospective nature might have led to information bias associated with the medical records. Second, the present study was a single-center investigation, which may have led to population bias. Overcoming these biases may require prospective and/or multi-center studies. Third, although the criteria for minimum follow-up duration was set for three months, the postoperative timepoint at which the outcomes were determined was not standardized.

## Conclusion

We performed a single-center cohort study to evaluate the effectiveness of surgery and HBO therapy for ARONJ, along with a subgroup analysis by disease stage. Our findings suggest that extensive surgery is highly effective against ARONJ, regardless of disease stage, if there is a sequestrum separation and systemic tolerance, while HBO therapy before and after the surgical approach can be effective for some patients. Conservative surgery may be a useful option in situations in which extensive surgery cannot be applied to patients with poor general condition, particularly, if there is no sequestrum separation. Our cohort study provides additional rationalization for surgery and HBO in the treatment of ARONJ. Therefore, further studies are needed to identify therapeutic interventions for patients with treatment-refractory ARONJ who may have to endure long-term HBO therapy with the expectation of sequestrum separation.
